# Genome-Wide Detection of CNVs and Their Association with Meat Tenderness in Nelore Cattle

**DOI:** 10.1371/journal.pone.0157711

**Published:** 2016-06-27

**Authors:** Vinicius Henrique da Silva, Luciana Correia de Almeida Regitano, Ludwig Geistlinger, Fábio Pértille, Poliana Fernanda Giachetto, Ricardo Augusto Brassaloti, Natália Silva Morosini, Ralf Zimmer, Luiz Lehmann Coutinho

**Affiliations:** 1 Animal Biotechnology Laboratory, Animal Science Department, University of São Paulo (USP)/Luiz de Queiroz College of Agriculture (ESALQ), Piracicaba, São Paulo, Brazil; 2 Embrapa Pecuária Sudeste, São Carlos, São Paulo, Brazil; 3 Institute of Bioinformatics, Department of Informatics, Ludwig-Maximilians-Universität München (LMU), Amalienstrasse 17, 80333, München, Germany; 4 Embrapa Informática Agropecuaria, Campinas, São Paulo, Brazil; University of Bologna, ITALY

## Abstract

Brazil is one of the largest beef producers and exporters in the world with the Nelore breed representing the vast majority of Brazilian cattle (*Bos taurus indicus*). Despite the great adaptability of the Nelore breed to tropical climate, meat tenderness (MT) remains to be improved. Several factors including genetic composition can influence MT. In this article, we report a genome-wide analysis of copy number variation (CNV) inferred from Illumina® High Density SNP-chip data for a Nelore population of 723 males. We detected >2,600 CNV regions (CNVRs) representing ≈6.5% of the genome. Comparing our results with previous studies revealed an overlap in ≈1400 CNVRs (>50%). A total of 1,155 CNVRs (43.6%) overlapped 2,750 genes. They were enriched for processes involving guanosine triphosphate (GTP), previously reported to influence skeletal muscle physiology and morphology. Nelore CNVRs also overlapped QTLs for MT reported in other breeds (8.9%, 236 CNVRs) and from a previous study with this population (4.1%, 109 CNVRs). Two CNVRs were also proximal to glutathione metabolism genes that were previously associated with MT. Genome-wide association study of CN state with estimated breeding values derived from meat shear force identified 6 regions, including a region on BTA3 that contains genes of the cAMP and cGMP pathway. Ten CNVRs that overlapped regions associated with MT were successfully validated by qPCR. Our results represent the first comprehensive CNV study in *Bos taurus indicus* cattle and identify regions in which copy number changes are potentially of importance for the MT phenotype.

## Introduction

*Bos taurus* is a well-studied model organism [1,2] and a species of great agricultural relevance, especially for Brazil, which is one of the world’s largest beef exporter [3] with a herd of estimated 209,541 million head [4]. Nelore (*Bos taurus indicus*) is the main cattle breed in Brazil [5] and is, like most *Bos taurus indicus* subspecies, adapted to the tropical Brazilian climate. However, meat tenderness (MT) of Nelore is not comparable to taurine breeds (*Bos taurus taurus*) [6].

The MT phenotype was first investigated in the 1920s [7], and is already well-studied for several livestock species [8–13]. An established measure of MT is shear force, and *Bos taurus indicus* typically requires a higher shear force compared with *Bos taurus taurus* to disrupt the beef fibers [14]. Larger muscle fibers, cross-bridges between filaments and reduced myofibrillar proteolysis are features that influence MT of indicus breeds [15,16]. Stress induced by genetic or environmental sources is also known to negatively affect MT [17].

Structural genetic variation associated with traits of interest are promising targets for animal breeding [18]. Copy number variation (CNV) is one of the frequently observed structural genomic variations and is, thus, increasingly being studied in cattle [19–28]. CNVs are defined as large genomic regions (conventionally >1 kb) with deviation from the normal diploid state due to duplication or deletion events [29]. CNVs are associated with several important phenotypes in humans [30–32] and livestock animals [33–35]. In cattle, chronic interstitial nephritis [36] as well as osteopetrosis [37] and birth defects [38] have been previously associated with CNVs. A CNV in the gene encoding CASL-like protein 2 (*MICAL-L2*), which performs a critical role in the development of muscle fibers, is correlated with gene expression in cattle making it an important molecular marker [39].

Experimental detection of CNVs on a large scale can be done using comparative genomic hybridization (CGH, [40]) or next-generation sequencing (CNV-seq, [41]). Although these are the most accurate methods to detect CNVs, they are still expensive to apply on many samples [42]. Thus, several studies in cattle inferred CNVs from SNP-chip data with subsequent validation of selected regions by quantitative polymerase chain reaction (qPCR) [19–28].

In this article, we infer CNVs in the genome of Nelore cattle, concatenate them into CNV regions (CNVRs) and validate a subset with qPCR. In addition, we perform a genome-wide association study of CN state with a quantitative MT phenotype derived from meat shear force. We identify MT-related genes and quantitative trait loci (QTLs) overlapping new and previously reported cattle CNVRs to establish relationships between structural genomic variation and MT.

## Results

### A genome-wide map of CNV regions in Nelore

**Fig 1:**
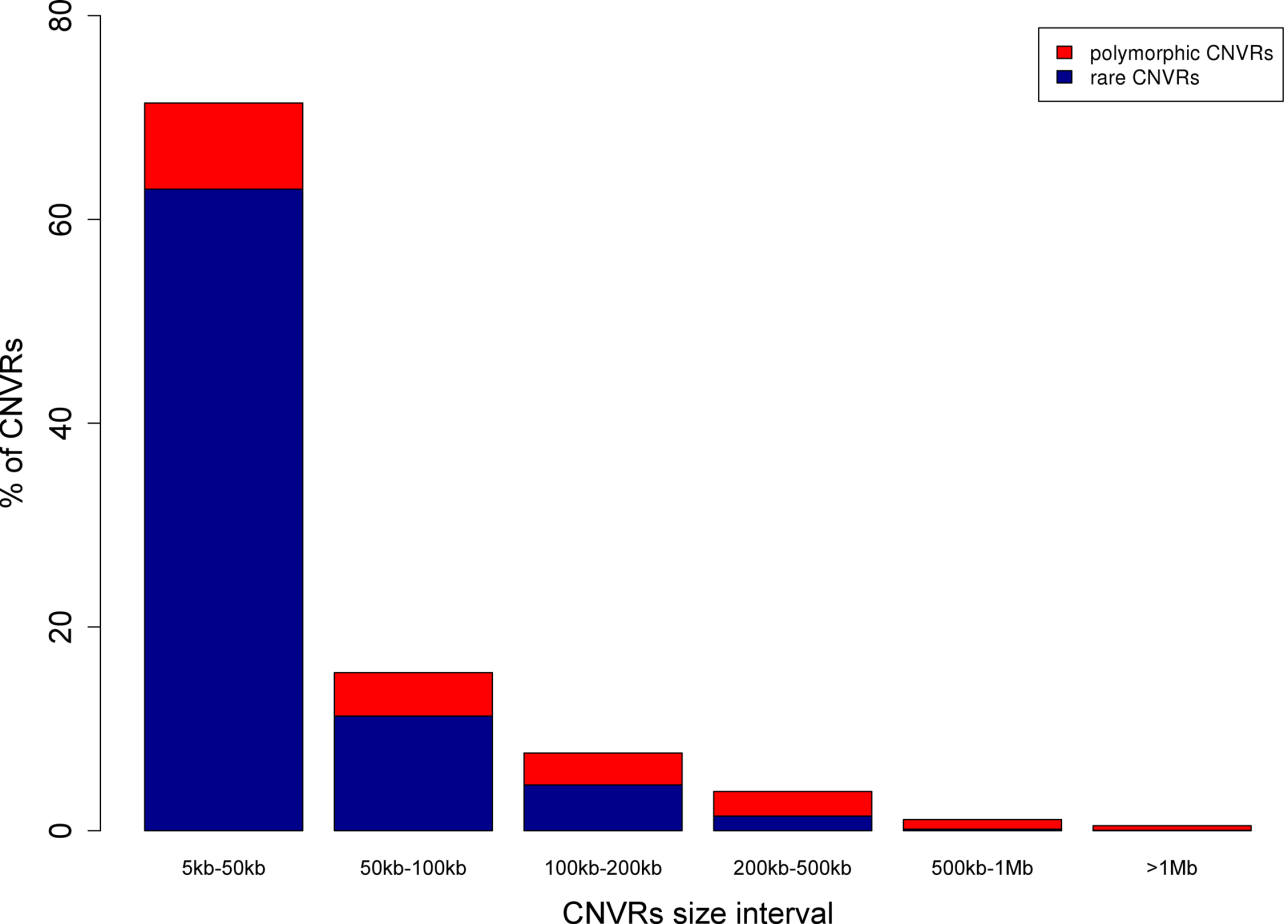
Distribution of CNVR length.

**Fig 2:**
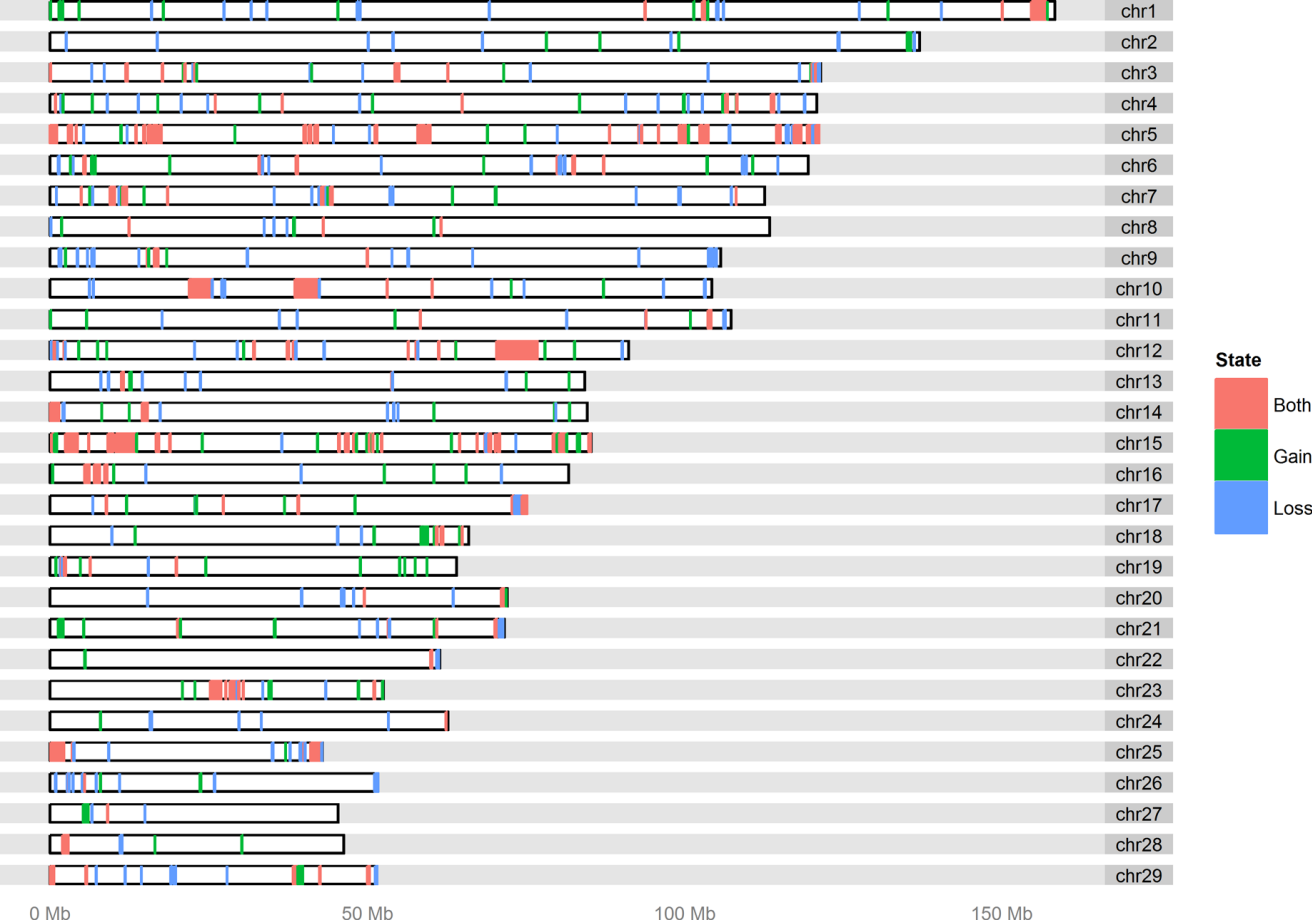
Chromosomal distribution of 521 polymorphic CNVRs (>1% of the population). The regions were categorized into loss or gain of copy number and states for which both events were observed.

We used SNP genotype data from 723 male Nelore animals to infer 49,997 CNV calls and 2,649 CNVRs using PennCNV [43] and CNVRuler [44], respectively (see Materials and Methods for genotyping, CNV calling and CNVR concatenation). The CNVRs represent 6.5% of the *Bos taurus* genome (170.6 Mb, genomic positions listed in S1 Table). The chromosomal proportion covered by CNVRs varies between chromosomes (from 2.3% to 19.7% for BTA22 and BTA15, respectively). The number of regions with copy loss and gain were 1,454 and 891, respectively. Presence of both types occurred in 304 regions. Average CNVR size was 64.4 kb, ranging from 5 kb (minimum threshold for CNV calls, see Materials and Methods) to 4.3 Mb. For each CNVR, the relative frequency of animals with an overlapping CNV ranges from 0.1% (1 out of 723) to 99.8% (722 out of 723, S1 Table). CNVRs with size between 5 and 50 kb represent the majority of our findings (71.4%) whereas CNVRs larger than 1 Mb were rarely observed (0.5%, Fig 1). We found 521 CNVRs to occur in more than 1% of the population and denote them as ‘polymorphic CNVRs’ in the following. These regions represent 3.2% of the *Bos taurus* genome (86.4 Mb, Fig 2). To exclude the possibility that polymorphic CNVRs with high frequency (occurring in >75% of our population) are technical artifacts of the mapping of indicus data onto the taurus assembly, we checked whether these CNVRs contained exclusively events of a particular CNV state (indicating rather genomic differences between taurus and indicus than individual CNVs within indicus). However, we did not find cases for which >95% of the contained CNV calls displayed the same CNV state, arguing against false positive detections due to the mapping.

**Fig 3:**
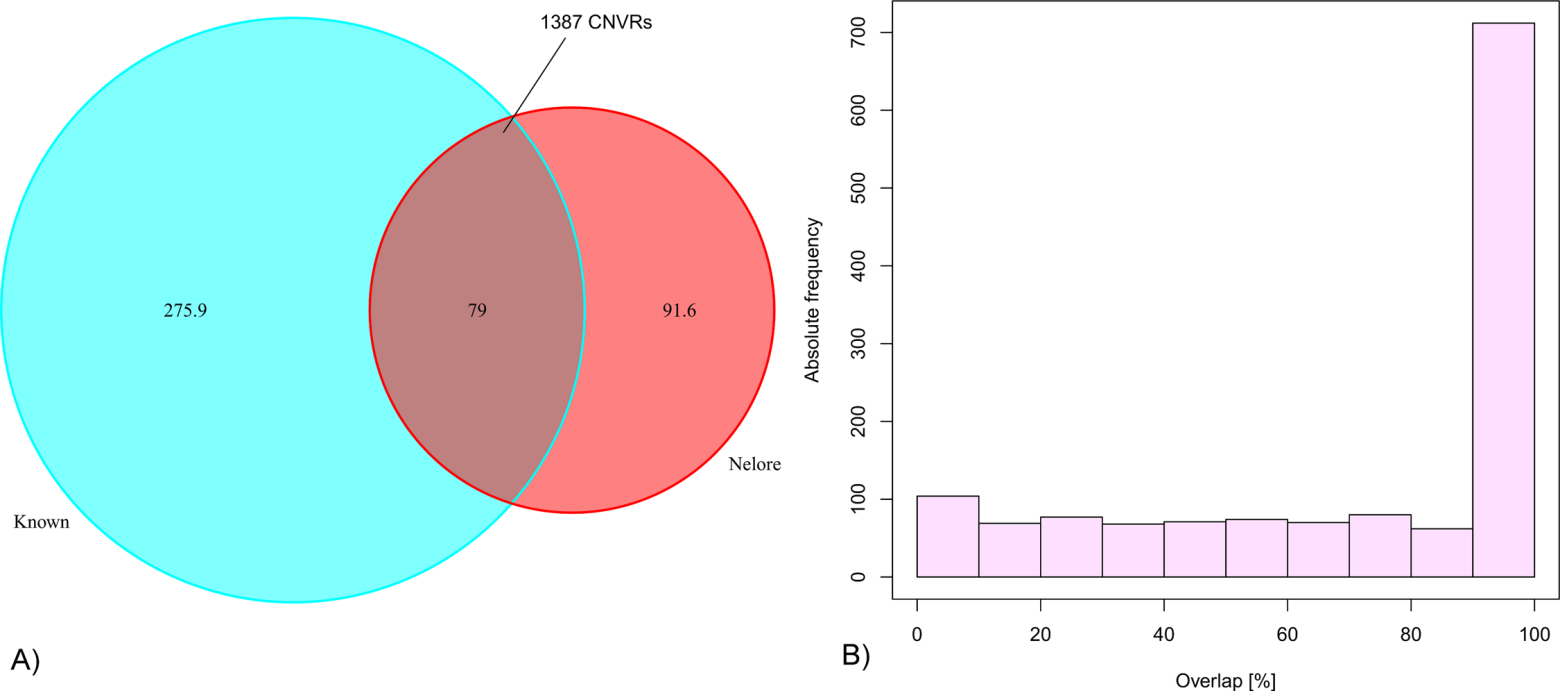
Overlap of the detected Nelore CNVRs with known cattle CNVRs. **A)** The Venn diagram shows the genomic size in Mb that is overlapped. Known CNVRs listed in S3 Table were reduced to unique and non-overlapping CNVRs. The overlapped fraction of 79 Mb corresponds to 1,387 Nelore CNVRs. **(B)** For these 1,387 CNVRs, the histogram shows the number of CNVRs (*y*-axis) overlapping with known CNVRs by the percentage shown on the *x-*axis. For example, genomic locations of >700 Nelore CNVRs overlap individually >90% with genomic locations of known CNVRs.

When comparing our CNVRs to previously reported cattle CNVRs (denoted herein as ‘known CNVRs’; see S2 Table and Materials and Methods for details), we found 1,387 (52.3%) overlapping regions. The total overlap corresponds to 79 Mb (46.3%) of the genomic area covered by the Nelore CNVRs (Fig 3). Repeated sampling of random genomic regions, matching our CNVRs in size and chromosomal distribution, showed that the overlap with known CNVRs is significantly larger than expected by chance (permutation *p*-value < 0.001, Material and Methods and S5 Table).

As different platforms can yield different results, we specifically compared the Nelore CNVRs with CNVRs previously obtained using the same High Density Illumina SNP chip [23]. We found 1,048 overlapping Nelore CNVRs, accounting for 53.9 Mb (31.6%) of the genomic area covered by the Nelore CNVRs (S5 Fig).

#### Association of CNVs with biological functions and processes

**Fig 4:**
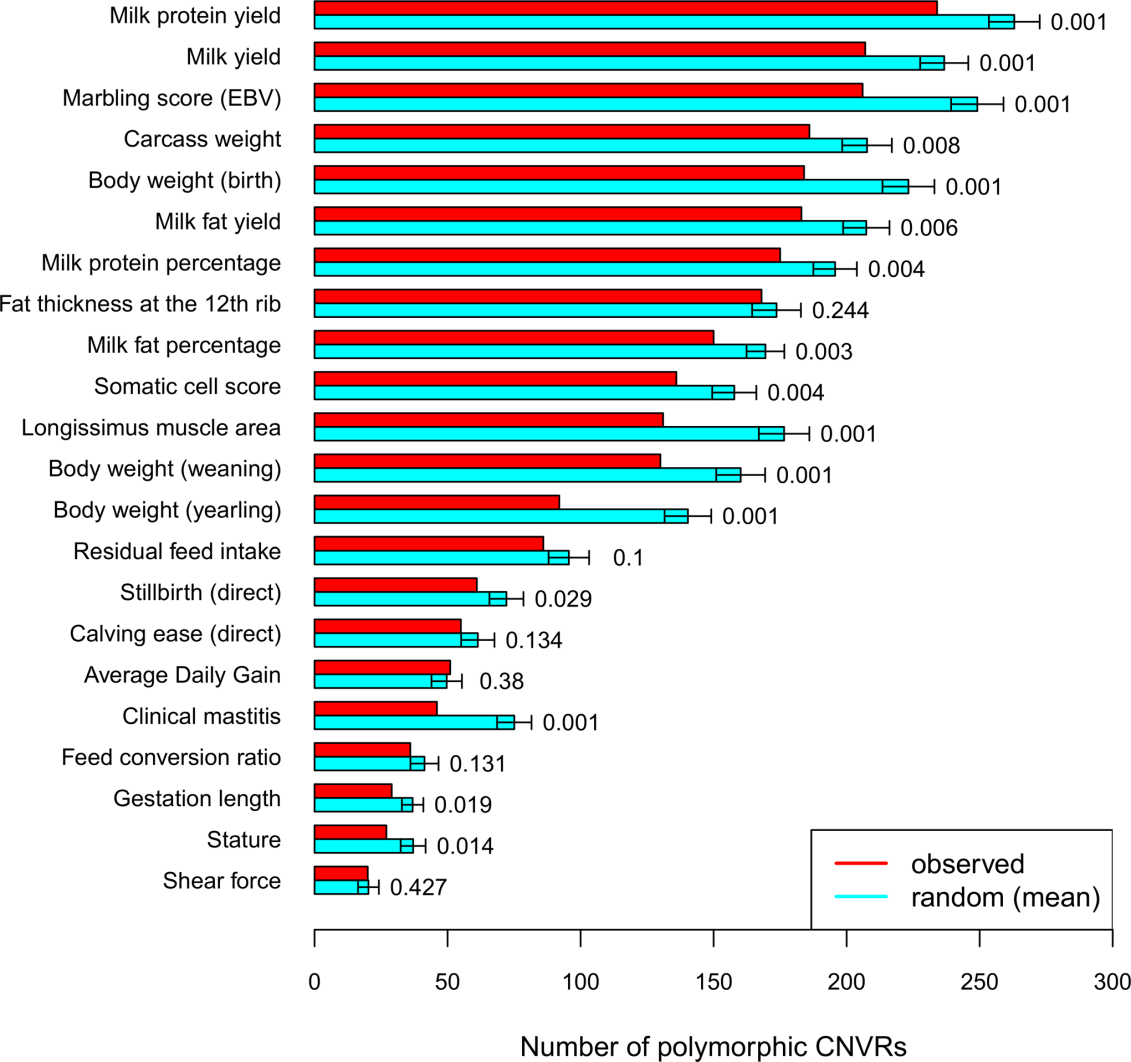
QTL-traits that overlap with polymorphic CNVRs in the Nelore population. Depicted are the most frequently overlapped traits. Red bars correspond to the observed number of polymorphic CNVRs overlapping with the respective trait, and cyan bars indicate the mean overlap when sampling 1000x random regions matching the polymorphic CNVRs in size and chromosomal location. The error bars indicate the standard deviation and permutation *p-*values are listed on the right.

We found 2,531 Nelore CNVRs (95.5%) overlapping at least one base with QTLs from Cattle Animal QTLdb [45] (genomic positions in S4 Table). However, most of the cases are CNVRs residing (completely overlapped) in a QTL region (S1 Fig). A total of 482 polymorphic CNVRs overlapped with 2,310 QTLs, corresponding to 282 traits including several milk-related phenotypes (Fig 4). Regarding meat quality, marbling score (intramuscular fat) is frequently found among those overlapped QTL traits. Despite the presence of CNVRs in QTL regions, this is not as common as expected given that genome coverage of QTLs is large and not well defined for cattle. Compared to randomly sampled genomic regions the detected CNVRs overlap significantly less (permutation *p-*value < 0.001) with the QTLs than expected by chance (Material and Methods and S5 Table). The same tendency was observed for the individual traits depicted in Fig 4.

Genomic positions of 44% of our CNVRs overlapped with 2,750 different genes (S6 Table), which were found most frequently inside CNVRs (69.2%, S1 Fig). On the other hand, 64 genes are overlapped by more than one CNVR. As observed for cattle QTLs, overlaps between CNVRs and genes were not as common as expected. We found that in comparison to randomly sampled regions the detected CNVRs overlap significantly less (permutation *p-*value < 0.001) with the annotated genes than expected by chance (Material and Methods and S5 Table). As depicted in Table 1, we found genes overlapping with polymorphic CNVRs enriched for immunological functions (including the following gene sets: major histocompatibility complex, antigen processing and presentation, and immunoglobulin). A similar observation has been reported for other mammals, where immunological genes were enriched inside CNVRs [46–49] and associated with susceptibility to diseases [50,51].

**Table 1:** Functional groups associated with polymorphic CNVRs (>1% of the population). The statistical significance level was set to 0.01. The enrichment *p*-values listed in the third column were computed using DAVID [62].

**GO terms**		
**GO ID**	**Description**	***P-*value**
GO:0004984	Olfactory receptor activity	1e-23
GO:0007186	G-protein coupled receptor protein signaling pathway	3.5e-15
GO:0007166	Cell surface receptor linked signal transduction	4.7e-12
GO:0016021	Integral component of membrane	2.5e-06
GO:0031224	Intrinsic component of membrane	2.7e-06
GO:0019882	Antigen processing and presentation	4.5e-06
GO:0042611	MHC protein complex	0.000011
GO:0032561	Guanyl ribonucleotide binding	0.00018
GO:0019001	Guanyl nucleotide binding	0.00022
GO:0042612	MHC class I protein complex	0.00059
GO:0005525	GTP-binding	0.0018
GO:0050909	Sensory perception of taste	0.0024
GO:0002474	Antigen processing and presentation of peptide antigen via MHC class I	0.0025
GO:0005833	Hemoglobin complex	0.0076
GO:0007606	Sensory perception of chemical stimulus	0.0087
**Protein sets**		
**Protein set ID**	**Description**	***P-*value**
IPR003597	Immunoglobulin C1-set	6.1e-06
IPR007110	Immunoglobulin-like domain	0.000012
IPR003006	Immunoglobulin/major histocompatibility complex, conserved site	0.000026
IPR013783	Immunoglobulin-like fold	0.000086
IPR010579	MHC class I, alpha chain, C-terminal	0.00018
IPR011161	MHC class I-like antigen recognition	0.001
IPR001400	Somatotropin hormone	0.0016
IPR007960	Mammalian taste receptor	0.0016
IPR018116	Somatotropin hormone, conserved site	0.0021
IPR013106	Immunoglobulin V-set domain	0.0036
IPR002338	Haemoglobin, alpha	0.0038
IPR001039	MHC class I, alpha chain, alpha1 and alpha2	0.004
IPR001461	Propeptide, peptidase A1	0.0099

Olfactory receptors (OR) were among the significantly enriched GO terms (DAVID enrichment *p-*value = 1e-23). This is again in agreement with previous studies, in which the OR gene family was found enriched in mammalian CNVRs [52–55], and in cattle CNVRs in particular [21,24–28,56–58]. Biological functions involving GTP are also significantly enriched, which is consistent with previous findings in cattle [59,60] and goat [61].

#### Association of CNVs with MT

MT-related QTLs from the Cattle Animal QTLdb (“Tenderness score” and “Shear force”) fell into 236 of the detected 2649 CNVRs (8.9%, corresponding to 18.2 Mb, S4 Table). The genes overlapping those CNVRs are enriched for peptidase and protease related pathways (*Aspartic peptidase*, *N-terminal*: IPR012848; *Aspartic peptidase*, *active site*: IPR001969, *aspartic-type peptidase and endopeptidase activity*: GO:0070001 and GO:0004190).

Additionally, we found that 75 out of the 132 (56.8%) Nelore-specific QTLs associated with shear force (measured at 0, 7 and 14 days after slaughter in a previous study conducted with the same population [63]) overlapped with 109 of our Nelore CNVRs (24 polymorphic, S7 Table). We also found the CNVRs to overlap unexpectedly less with the Nelore QTLs compared to randomly sampled regions (only 56 out of 1000 permutations resulted in an overlap as small or smaller as the observed one, corresponding to a permutation *p-*value of 0.057, Material and Methods and S5 Table). Similar to the findings for QTLs from the cattle QTLdb, we observed that overlapping CNVRs locate predominantly within the Nelore-specific QTLs (S1 Fig).

We validated 10 MT-related CNVRs using qPCR (see Material and Methods). The concordance of copy number state (loss, normal and gain) between qPCR and SNP-array results was in average 66.4%, ranging from 46% to 86% of samples for each region tested (S8 Table). The inferred copy number state (from 0n to 4n) was calculated as a normalized ratio of PCR cycle threshold values (Material and Methods, S2 Fig and S9 Table).

**Fig 5:**
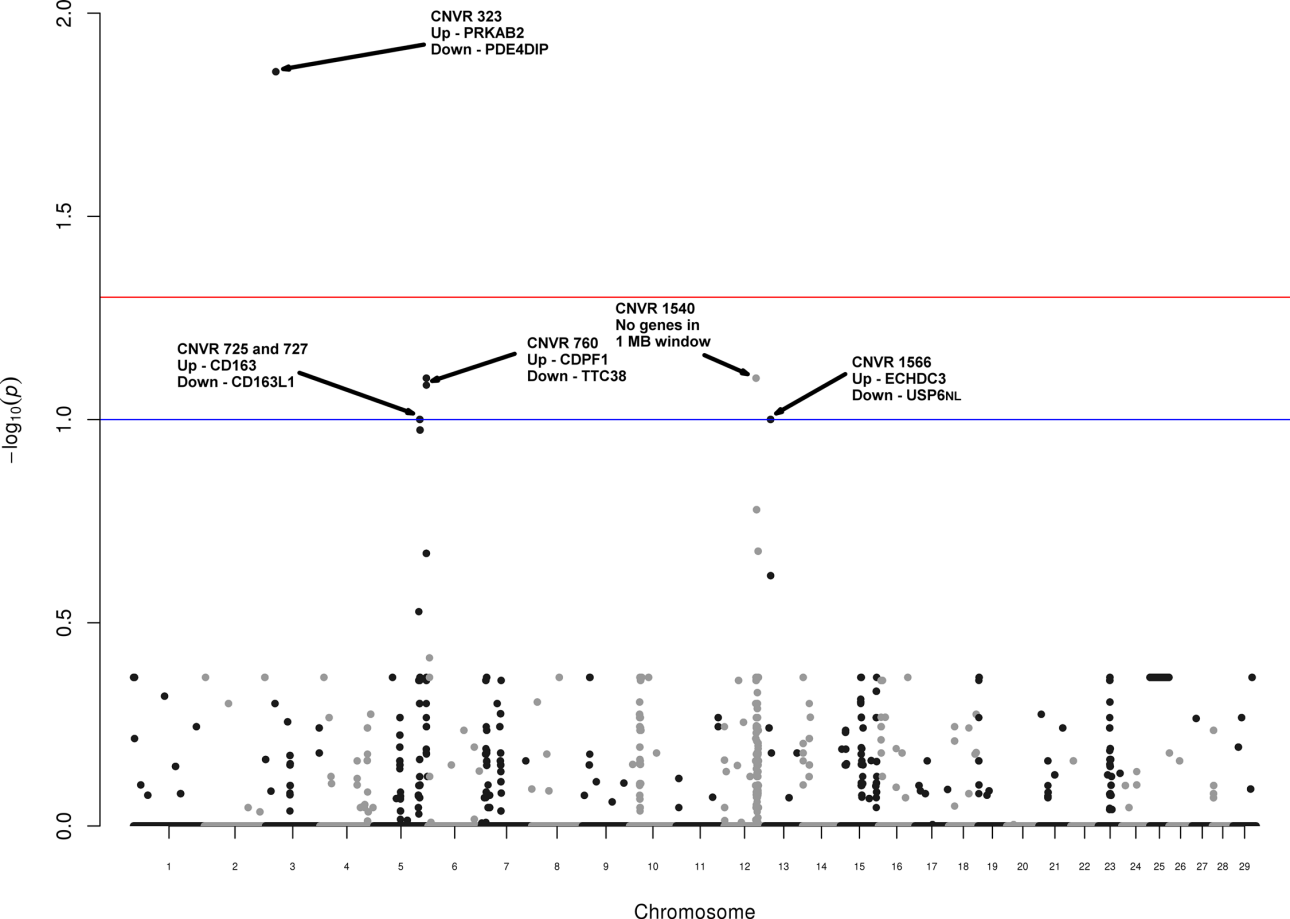
Genome wide association of CNV state with shear force estimated breeding value (SF-EBV). Manhattan plot for 447 CNV segments on somatic chromosomes (*x*-axis) and the corresponding -log_10_ *p-*value (*y*-axis) indicating the association strength with SF-EBV. Multiple testing correction was performed, controlling the false discovery rate (FDR, [64]). Red and blue lines indicate FDR-corrected *p-*values of 0.05 and 0.1, respectively. The black arrows indicate CNVRs with *p-*value <0.1 and list the nearest protein-coding genes up- and downstream of the segment (in a genomic window ≤1Mb). Raw signal strength is shown in S3 Fig.

To test the genome-wide association of the detected CNVs with MT, we used estimated breeding values derived from meat shear force (SF-EBV) as the quantitative MT phenotype (see Material and Methods for details). As the CNVRs are a result of concatenated individual CNVs and can thus contain different CN states, we constructed CNV segments from subsequent probes with highly similar genotype (constructed without parental information, see Material and Methods for details). This yielded 447 CNV segments (S11 Table) constructed from 3,242 probes (S12 Table) that deviate from the 2n state in ≥5% of the population. The number of probes in each segment ranged from 1 to 9, with 7.25 probes on average. The association level for each segment after multiple testing correction is depicted in Fig 5.

Considering the FDR-corrected *p*-values depicted in Fig 5, we found a significantly associated CNV segment inside CNVR 323 (association *p-*value = 0.014). Gain state in CNVR 323 was validated by qPCR in 3 of 6 samples (S8 Table). Additional candidates were found when ranking all 447 CNV segments by FDR-corrected *p*-value and relaxing the significance level. This yielded 4 CNV segments located in CNVRs 725, 727, 1540, 1566, respectively, and 2 CNV segments located in CNVR 760 with association *p*-value <0.1. Although a significance level of 0.1 can be considered anti-conservative from a statistical point of view, we further investigated the additional candidates and checked whether they coincide with the aforementioned CNVRs validated by qPCR. CNVR 760 and 1566, containing 3 of the 6 candidate segments, were tested and successfully validated by qPCR in 23 of the 30 sires (i.e. correspondence >75%, S8 Table).

**Fig 6:**
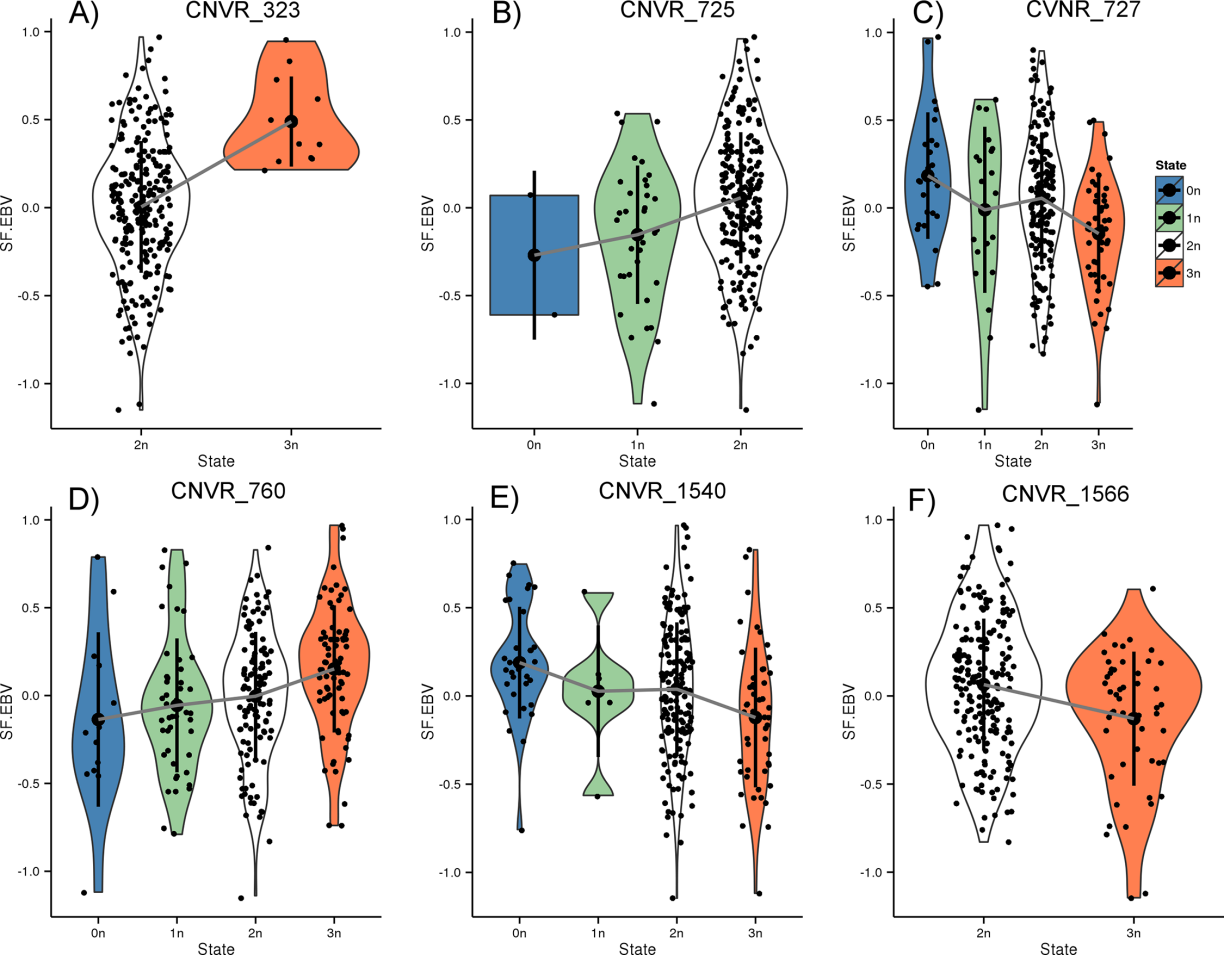
Shear force estimated breeding value (SF-EBV) distribution in each CN state for six significant CNV segments. Each dot represents an animal in the corresponding CN state (0-3n) on the *x*-axis and the assigned SF-EBV on the *y*-axis. The legend on the top right displays the color code for the CN state. See the main text for a detailed description of each segment and S11 Table for additional details.

Fig 6 shows the SF-EBV distribution stratified by observed CN state for the significantly associated segment in CNVR 323 as well as the candidate segments in the corresponding 5 CNVRs (for CNVR 760, the higher ranked segment was selected). Four of the segments correspond to annotated Ensembl CNVRs. It has been previously reported that the expression of genes up to 450 kb distal from CNVR boundaries can be affected [65]. Based on that, we investigated a genomic window 450 kb up- and downstream, which corresponds to a 900 kb ≈ 1 Mb window, and found a total of 32 protein-coding genes to be located in the vicinity of the segments.

The CNV segment in CNVR 323 is located on BTA3 and CN is positively correlated with SF-EBV, i.e. increase in CN is associated with genetic potential for tougher meat. Interestingly, we found the region to be an Ensembl gain CNVR with several adjacent protein-coding genes (S4 Fig). The same tendency is observed for the segments in CNVR 725 and CNVR 760 on BTA5, i.e. CN is positively correlated with SF-EBV. In addition, the segment in CNVR 725 matches an Ensembl CNVR and is highly similar to a previously described CNVR (94% overlap, S3 Table).

On the other hand, the segments in CNVR 727, 1540, and 1566 located on BTA5, BTA12, and BTA13, respectively, show the reverse tendency, i.e. CN is negatively associated with SF-EBV. This indicates that for these regions, increase in copy number is associated with genetic potential for tender meat. CNVR 1566 coincides with an Ensembl CNVR and is highly similar to a previously described CNVR (97% overlap, S3 Table).

## Discussion

In recent years, the main genetic variant investigated in genome-wide association studies is single nucleotide polymorphism (SNP); however, the extension to larger regions of variation such as CNVs is feasible and beneficial [66,67]. To investigate structural variation in the Nelore breed, we applied a computational approach and inferred a genome-wide CNV map from SNP-chip genotyping data. Our results revealed that variable regions are scattered across the genome and represent a large portion of it (6.5%), as reported for other cattle breeds [19,28].

Comparison of CNVs among studies, even within the same species, is not trivial as their mapping is typically based on different reference genomes, and the exact genomic start and end coordinates (CNV boundaries) are subject to biological and technical variation [57,68,69]. CNV studies in *Bos taurus* reported CNVRs that are predominantly based on the UMD_3.1 and Btau_4.0 reference assemblies. Conversion of genomic coordinates resulted in considerable data loss, as we could not convert 36.3% of the CNVRs from Btau_4.0 to UMD_3.1 using liftOver [70]. Nevertheless, we found significantly more CNVRs to overlap with known CNVRs than expected by chance (permutation p-value < 0.001), indicating that a considerable fraction of CNVRs is conserved between Nelore and other cattle breeds.

Genes and QTLs are important functional regions of the genome and are thus not expected to be subject to wide-range rearrangements, such as CNVs. This is in agreement with our finding that Nelore CNVRs overlap less frequently with genes and QTLs than random regions of the genome. Therefore, CNVRs located in genes and QTLs are of special interest. Several polymorphic CNVRs (found in more than 1% of the population) overlap with MT-QTLs from cattle QTLdb [45]. The enriched peptidase and protease gene families at these regions have a remarkable role in post-mortem tenderization as they are involved in MT-associated proteolysis and connective tissue turnover [71]. Investigation of genes at Nelore MT-QTLs for this population [63] overlapping our CNVRs showed an enrichment for immunological functions. A notable exception to immunological genes is the diaphanous-related formin *3* gene (*DIAPH3*), which is overlapped by two polymorphic CNVRs. Actin-based processes are regulated by diaphanous-related formins [72] and influence MT-associated proteolysis [73]. Moreover, diaphanous-related formins affect specific GTPases [74], which showed a different mRNA expression level between animals with tough and tender meat [60].

We also found enriched processes involving GTP (Table 1, *G-protein coupled receptor protein signaling pathway* and *GTP binding*). Previous MT and skeletal muscle studies described the influence of GTP-related genes on myotube morphology, skeletal muscle myogenesis, regeneration and calcium physiology [75–78]. The impact on calcium content is especially notable as it affects MT through postmortem protein degradation in skeletal muscle [79,80]. A representative example is the proteolysis-associated ATP/GTP binding protein-like 3 gene (*AGBL3*), present in fewer copies in Korean beef cattle (Hanwoo) when compared to Holstein dairy breed [59]. As beef and dairy breeds differ in tenderness [81], further investigation is desired on whether CNV in the AGBL3 locus affects MT. In Nelore, we found a region of polymorphic CNV (CNVR_507) 96 kb downstream of *AGBL3*.

The GTP-binding protein RAD-like gene is a validated meat quality biomarker [82], and GTP-related genes are also targets of differentially expressed miRNAs between MT phenotypes [83]. As the number of miRNA-binding sites is significantly higher inside CNVRs [84], this indicates a potential regulatory mechanism that could modulate transcripts synthesized at CNVRs.

To detect CNV events linked with MT, we performed a genome-wide association analysis between SF-EBV and CNV segments composed of highly similar probes, which are thus identical by state (IBS) and not necessarily by descent (IBD) as parental information was not available. The CNV segments associated with SF-EBV did not overlap QTLs previously found in the same population ([63], see S7 Table) indicating that GWAS studies using only SNP association may miss important QTL associated with CNV state. Exclusion of relevant CNV probes from the SNP-GWAS due to deviation from Hardy-Weinberg equilibrium and stringent multiple testing correction (with a typically conservatively chosen significance level) are likely causes for that.

Among the resulting significantly associated segments, we found segments located in the vicinity of cAMP and PKA-related genes. In addition, the small GTP-binding protein GTPase RAC1 (located in CNVR 2447) interacts directly with cAMP-dependent protein kinase A (PKA) [85]. The detected statistically significant CNV segment on chromosome 3 is located 87 kb upstream of PDE4DIP, a gene involved in cAMP and cGMP signaling [86]. It has been reported that cGMP signaling is involved in muscle contraction mechanisms [87], and cAMP signaling influences hypertrophy, metabolism and regeneration of skeletal muscle [88]. Located 150 kb upstream of the significantly associated CNV segment on chromosome 3 is *PRKAB2*, a subunit of the AMP-activated protein kinase. Presence of this kinase stimulates calcium transportation in sarcolemma [89] and affects calpastatin gene transcription and protein phosphorylation [90]. The Calpain-calpastatin system is known to be of key importance for postmortem proteolysis and meat tenderization [91].

The two CNV segments of CNVR 725 and 727, for which we found significant association between CN state and SF-EBV, are located in close proximity to *CD163* and *CD163L1* (located within CNVR 725). These genes influence the intra-cellular hemoglobin level [92] known to be associated with the density of cross-sectional muscle areas [93]. Interestingly, we also found the hemoglobin complex (GO:0005833) and hemoglobin alpha (IPR002338) enriched in genes overlapped by polymorphic CNVRs. Hemoglobin homeostasis is tightly linked to carbon monoxide (CO) oxidative stress signaling [94,95]. CO induces cGMP and antioxidant agents such as reduced gluthathione [95]. We also found two polymorphic CNVRs (CNVR 2061 and 2443) that overlapped two QTLs for glutathione redox balance [96], which is an interesting case for further targeted investigation as the glutathione dimer has been reported as a MT-predictor [97]. Moreover, those QTLs are overlapped by the gene encoding SMAD specific E3 ubiquitin protein ligase 1 (*SMURF1*), which inhibits the transduction pathway of myostatin [98]. The gene encoding chaperonin containing TCP1, subunit 6B (*CCT6B*) was also mapped to the glutathione redox balance QTLs [96] and overlapped with polymorphic CNVRs. Chaperonins play a crucial role in the protein folding process [99], which is in turn of key importance for meat tenderization [8].

The enriched *Somatotropin hormone* (Table 1) is significantly involved in growth and differentiation of skeletal muscle [100]. Interestingly, the gene encoding Insulin-like growth factor 2 hormone (*IGF2*) was found inside the polymorphic CNVR 2647. IGF2 is also a predicted target of the bta-let-7b miRNA (MI0005453), found inside another polymorphic CNVR (CNVR 760), which contains two CNV segments associated with SF-EBV. Notably, *IGF2* is a known stimulator of myoblast differentiation [101,102] and reduced *IGF2* expression is associated with improved tenderness [103].

The supplemental discussion (S1 Text) contains further examples and also discusses influences on genes frequently reported to be associated with MT such as μ-calpain and calpastatin (*CAPN1* and *CAST*, [104–107]) and certain heat shock proteins (HSPs, [108,109]). However, they did not overlap with Nelore-specific CNVRs reported here.

In conclusion, we conducted the first comprehensive CNV study in *Bos taurus indicus* cattle and found that a considerable portion of the Nelore genome contains CNVRs. Concentrating on the MT phenotype, we systematically investigated the detected CNVRs with respect to the overlap with functional regions such as QTLs, the enrichment of gene functions, and the genome-wide association with MT. We identified several regions previously associated with MT as well as new regions potentially important for MT. We thus assume our results to serve as a good starting point for future studies on structural variations related with MT and skeletal muscle physiology.

## Materials and Methods

### Ethical statement

All experimental procedures involving steers in this study were approved by the Institutional Animal Care and Use Committee Guidelines (IACUC) from Brazilian Agricultural Research Corporation (EMBRAPA) and sanctioned by the president Dr. Rui Machado.

### Population and genotyping

The population under study comprises 777 Nelore males including 30 founding sires used to produce the population. For SNP-array analysis, genomic DNA samples were extracted from semen straws (sires) and blood (offspring). All animals were genotyped using Illumina Bovine HD Beadchip arrays (>770,000 SNP markers). The call rate (percentage of successfully genotyped SNPs for a given animal) threshold was >95%. We excluded 54 animals from the analysis, as they did not satisfy the CNV calling filter criteria described in the next section, and applied all subsequent analysis to the remaining population of 723 animals. Raw genotyping data is available upon request (requires a signed declaration of exclusive research purpose).

#### CNV calling

Individual CNVs were called using PennCNV [43]. PennCNV incorporates Log R Ratio (LRR) and B Allele Frequency (BAF), which denote the log2 ratio of R (normalized total intensity of 2 SNP alleles) and the frequency of allele B defined as the normalized intensity ratio for each SNP allele, respectively. Application of PennCNV yielded 766 animals for which at least one CNV was inferred. LRR values were corrected for genomic wave bias of SNP-arrays [110] via “*genomic_wave*.*pl -adjust”*, considering a genomic window of 1 kb (500 bp up- and downstream of each investigated SNP). Sexual chromosomes were excluded from the analysis. The Population Frequency of B Allele (PFB) file was generated using a subset of the same Nelore population (671 animals).

A total of 43 samples with >150 CNV calls (“-qcnumcnv 150”) were excluded as they were expected to be of low quality (http://penncnv.openbioinformatics.org/en/latest/misc/faq/) and thus inflate CNVR length due to concatenation of false positively detected CNVs. Similarly, CNV calls <5kb (“-length 5k”) were excluded as such regions seem to be less concordant with CGH [111] and confidential CNV inference from SNP arrays has been reported accordingly for >5kb [112]. Default settings were used for B allele frequency drift (BAF_drift) and quality control waviness factor (QC_WC). LRR standard deviation (LRR_SD) was set to <0.3 (“-*qclrrsd* 0.3”) as previously suggested [19,23,113,114].

#### CNVR compilation

Filtered individual calls from PennCNV were concatenated into CNV regions (CNVRs) using CNVRuler [44]. Genomic areas with density <10% were excluded ("recurrence 0.1"). The recurrence trims a CNVR based on its frequency to avoid false positive predictions. Additionally, the option "*Gain/Loss separated regions*" was used to compile CNVRs based on their genotype (gain or loss). Overlapping "gain" and "loss" CNVRs were merged into single regions to account for genomic regions in which both events can occur (*"*both" CNVRs).

#### Validation by Qpcr

Quantitative PCR was carried out for nine CNVRs in all 30 sires of the population. The CNVRs were selected because they contain SNPs that are associated with MT or are located near to MT-related QTLs and genes. Additionally, CNVR323 was validated by qPCR in 31 non-sire samples as this CNV was not present in the sires. To ensure specificity of the validation, we only considered 6 of 31 samples that were predicted in 3n state in the region covered by the primer amplicon. Primers were designed using Primer3plus [115] and quality testing was performed with NetPrimer [http://www.premierbiosoft.com/netprimer]. In the genomic region represented by the primer, the presence of SNPs from SNPdb [116] was checked against Ensembl-Biomart (http://www.ensembl.org/biomart/martview) [117]. To ensure qPCR accuracy, only primers that did not contain SNPs from SNPdb were synthetized.

All primers (S9 Table) were tested in serial dilutions of pooled genomic DNA to achieve optimal qPCR conditions. A qPCR solution of 10 μl was used consisting of 5.0 μl SYBR Green 2x (Roche®), 0.5 μl forward primer (5 mM), 0.5 μl reverse primer (5 mM) and 4.0 μl of genomic DNA (2.5 ng/μl). The qPCR steps were as follows: 1) 95°C for 5 min, 2) 40 cycles of 95°C and 51–60°C (primer-dependent), 3) 72°C for 10–15 seconds (primer dependent). Each animal was tested in technical triplicates carried out in Light cycle 480 (Roche®).

Cycle thresholds (log2 Ct) were corrected by primer mean efficiency as calculated with LinReg [118]. ΔCt denotes Ct of targeted region minus Ct of control region (primer pair targeting the *BTF3* gene, which has been used before as a control gene for CNV studies in cattle [19,26]). ΔΔCt was calculated as ΔCt from the animal to be tested minus ΔCt of a diploid (2n) control [119]. The control value was estimated based on the average value of ΔCt from all 2n animals (estimated with PennCNV).

Copy number was estimated from the normalized ratio (NR): 2x2^-(∆∆Ct)^ [26]. Copy number states were categorized as 1n (partial deletion), 2n (normal state), 3n (one copy gain), 4n (two copy gain) and 5n (gain of more than two copies) based on the geometric average of two states [119]. Lack of amplification was considered as 0n (complete deletion). In PennCNV, each CNV is typically assigned to one of four possible states: 0n (complete deletion), 1n (partial deletion), 2n (normal state), 3n (one copy gain), and 4n (two copy gains). As qPCR can result in >4n copies (see above), a frequently used simplification is to separate all states in just two CNV types: gain or loss [26,56]. Accurate CNV detection can be biased due to biological or technical variation [57,68,69]. Moreover, qPCR amplicons usually represent only a fragment of the CNVR. Hence, to consider a sample as concordant, we required it to be detected by PennCNV (≥1 CNV call) as well as qPCR (≥1 CNVR primer pair) in the same CNV type (loss or gain). CNVRs with ≥1 concordant sample were considered as validated.

#### Annotation (QTLs, genes and enrichment)

CNVRs were screened for overlap with cattle QTLs from cattleQTLdb [45], specific MT-QTLs in Nelore [63], genes annotated in Biomart-Ensembl [120], and 25,620 predicted target transcripts of 676 miRNAs listed in the Microcosm Targets database, version 5 [121].

Genes, which overlapped CNVRs, were subjected to an enrichment analysis with DAVID [62] to identify metabolic pathways that are predominantly associated with genes prone to CNV. The enrichment analysis was carried out for the subset of regions in which CNVs occurred in >1% of the population, defined as polymorphic CNVRs.

#### Comparison with previous studies

The detected CNVRs (UMD_3.1 genomic coordinates) were compared to CNVRs from 14 previous cattle studies [19,21–26,56–59,114,122,123] and structural variation data for bovine from Ensembl [117]. Genomic coordinates of 11 studies were converted from Btau_4.0 to UMD_3.1 using liftOver [https://genome.ucsc.edu/cgi-bin/hgLiftOver]. CNVRs from different studies that overlapped >70% with each other were considered as detected by both studies. CNVRs from external sources are denoted as known CNVRs in this article (listed in S10 Table).

#### Overlap statistics

Overlap analysis was carried out using the Bioconductor package regioneR [124]. The package implements a general framework for testing overlaps of genomic regions based on permutation sampling. We repeatedly sampled random regions (*N* = 1000 permutations) from the UMD_3.1 genome assembly matching size and chromosomal distribution of the detected CNVRs (including all 2649 CNVRs and, separately, the 521 polymorphic CNVRs). CNVRs >1 Mb were excluded as they represented extreme outliers. In every permutation, the overlap was recomputed with a) cattle QTLs from QTLdb, b) UMD_3.1 genes, c) Nelore-specific QTLs, and d) known CNVRs. For robustness, we checked two overlap measures: (i) number of random regions that overlapped a target region, and (ii) total genomic size in bp that was overlapped.

#### Genome-wide association study of CNV state with SF-EBV

The tenderness evaluation was performed by measuring the shear force (SF) [7] on day 7 and day 14 after slaughtering. The SF values were used to estimate genetic breeding values for meat tenderness (SF-EBV, detailed procedure in [109]). A total of 250 animals with SF-EBV values were used herein.

Association analysis was carried out as follows: **1)** Probes within CNVs were assigned to the corresponding number of copies (0n, 1n, 2n, 3n, or 4n). **2)** As common CN polymorphisms (CNPs, allele frequency ≥5%) can be represented as SNPs of equal frequency [112], we selected all genotyped probes that deviated from the normal state (2n) in ≥5% of the population. **3)** Genome-wide association analysis with the SF-EBVs was carried out using PLINK 1.07, using the corresponding functionality for CNP data encoded in a probe-by-probe fashion (http://pngu.mgh.harvard.edu/~purcell/plink/gvar.shtml, [125]). **4)** Assuming subsequent probes to frequently measure the same CNV event [66], we concatenated subsequent probes with identical genotype in ≥95% of our population to CNV segments. It should be noted here, that they are thus identical by state (IBS) and not necessarily by descent (IBD) as parental information was not available. **5)** The probe with most significant *p-*value was selected as representative for the segment. **6)** CNV segment *p*-values were corrected for multiple testing by controlling the false discovery rate [64].

## Supporting Information

S1 Fig Observed overlap types. Shown is the number of overlaps (y-axis) corresponding to a specific overlap type (x-axis). This is illustrated for all CNVRs (cnvrs) and polymorphic CNVRs (poly.cnvrs) and their overlap with genes annotated in the UMD_3.1 assembly, cattle QTLs from QTLdb [45] and Nelore MT-QTLs [63], and known CNVRs (as listed in S2, S3 and S10 Tables).
(TIFF)

S2 Fig Average normalized ratios (NR) of qPCR cycle thresholds with indicated standard deviation. Shown is the average for each copy number (CN) state for 16 tested primers (Nr. 2–17 in S9 Table). NR mean and standard deviation (SD) are 1.057 ± 0.295, 1.964 ± 0.281, 2.855 ± 0.291 and 3.827 ± 0.255 for 1n, 2n, 3n, and 4n, respectively. See Materials and Methods for details.
(TIFF)

S3 Fig Manhattan plot of raw association level (before multiple testing correction) between CNV state of 447 CNV segments and shear force estimated breeding value (SF-EBV). (TIFF)

S4 Fig Genes in the vicinity of the significantly MT-associated CNV segment in CNVR 323. **(A)** Genomic positions. **(B)** Previously described CNVR in the Ensembl genome browser (http://www.ensembl.org/Bos_taurus/Location/View?r=3%3A22796624-22815905).
(TIFF)

S5 Fig Overlap of the detected Nelore CNVRs with CNVRs from Hou et al., 2012b. **(A)** The Venn diagram shows the genomic size in Mb that is overlapped. The overlapped fraction of 53.9 Mb corresponds to 1048 Nelore CNVRs. **(B)** For these 1048 CNVRs, the histogram shows the number of CNVRs (*y-*axis) overlapping with CNVRs from Hou et al. by the percentage shown on the *x-*axis. For example, genomic locations of >400 Nelore CNVRs overlap individually >90% with genomic locations of CNVRs from Hou et al.
(TIFF)

S6 Fig QTL-traits that overlap with polymorphic CNVRs in the Nelore population. Depicted are the most frequently overlapped traits and the respective overlapped genomic size in Mb.
(TIFF)

S1 Table Genomic positions of the detected CNVRs in the Nelore population. (CSV)

S2 Table CNV studies in cattle. (PDF)

S3 Table Nelore CNVRs, which overlap known CNVRs from previous studies. (CSV)

S4 Table Nelore CNVRs, which overlap cattle QTLs. (CSV)

S5 Table Overlap statistics. Shown is the number of CNVRs that overlap genes annotated in the bosTau6 assembly, QTLs from cattle QTLdb [45], Nelore MT-QTLs [63], and known CNVRs (S2, S3 and S10 Tables). Shown in brackets is the total genomic size in Mb that is overlapped. The observed overlap is shown in the 2nd column as compared to the mean overlap of random regions in the 3rd column (with standard deviation, SD). Random regions were sampled repeatedly (N = 1000) matching the CNVRs in size and chromosomal location. See S1 Fig for observed overlap types and Material and Methods for random sampling of genomic regions. The alternative tested, i.e. whether the observed overlap is less or greater than expected by chance, is shown in 4th column. The corresponding *p*-value (rounded to 3 decimal places) is shown in the 5th column. A *p-*value < 0.001 denotes that none of the 1000 permutations yielded an overlap as extreme as it has been observed.
(PDF)

S6 Table Nelore CNVRs, which overlap annotated *Bos taurus* genes in the UMD_3.1 genome assembly. (CSV)

S7 Table Nelore CNVRs, which overlap Nelore QTLs associated with meat tenderness [63]. (CSV)

S8 Table Detailed result description for the 10 CNVRs validated by qPCR. All 30 sires (population founders) with it respective CNV calls compared with it respective CN states identified by qPCR analysis on the same CNVR. Sires with ≥1 CNV call as well as ≥1 primer pair with concordant CN state (loss or gain) were considered as validated. CNVR323 was subjected to qPCR vallidation in 31 non-sire samples as this CNV was not present in the sires, considering only the 6 samples that were predicted in 3n state in the region covered by the primer amplicon.
(CSV)

S9 Table Primers used for CNVR validation by qPCR. (PDF)

S10 Table Cattle CNVRs reported in previous studies. (CSV)

S11 Table Genomic location, FDR-corrected *p-*value, representative probe ID, number of animals for each state, and adjacent genes for the 447 CNV segments depicted in Fig 5. (CSV)

S12 Table Genotype concordance of the 3,242 CNV probes that were used to construct the 447 CNV segments listed in S11 Table. (CSV)

S1 Text Genes previously reported to influence meat tenderness. (PDF)
