# Supplementary figures and images for: Genome-Wide Detection of CNVs and Their Association with Meat Tenderness in Nelore Cattle
Source: PLoS One. 2016 Jun 27;11(6):e0157711. doi: 10.1371/journal.pone.0157711 (PMC4922624; doi:10.1371/journal.pone.0157711)

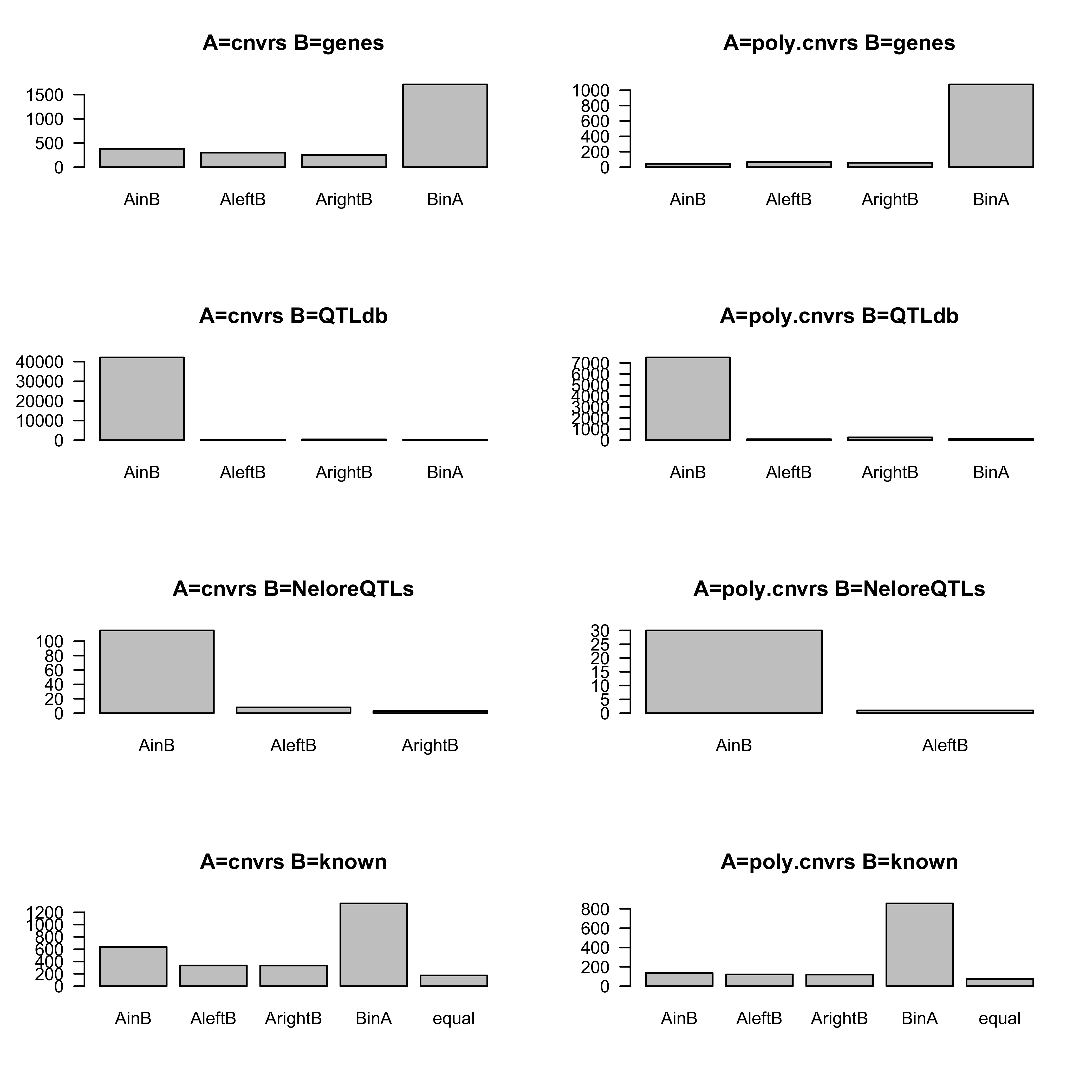

Supplement: S1 Fig — Shown is the number of overlaps (y-axis) corresponding to a specific overlap type (x-axis). This is illustrated for all CNVRs (cnvrs) and polymorphic CNVRs (poly.cnvrs) and their overlap with genes annotated in the UMD_3.1 assembly, cattle QTLs from QTLdb [45] and Nelore MT-QTLs [63], and known CNVRs (as listed in S2, S3 and S10 Tables). (TIFF) [file pone.0157711.s001.tiff]

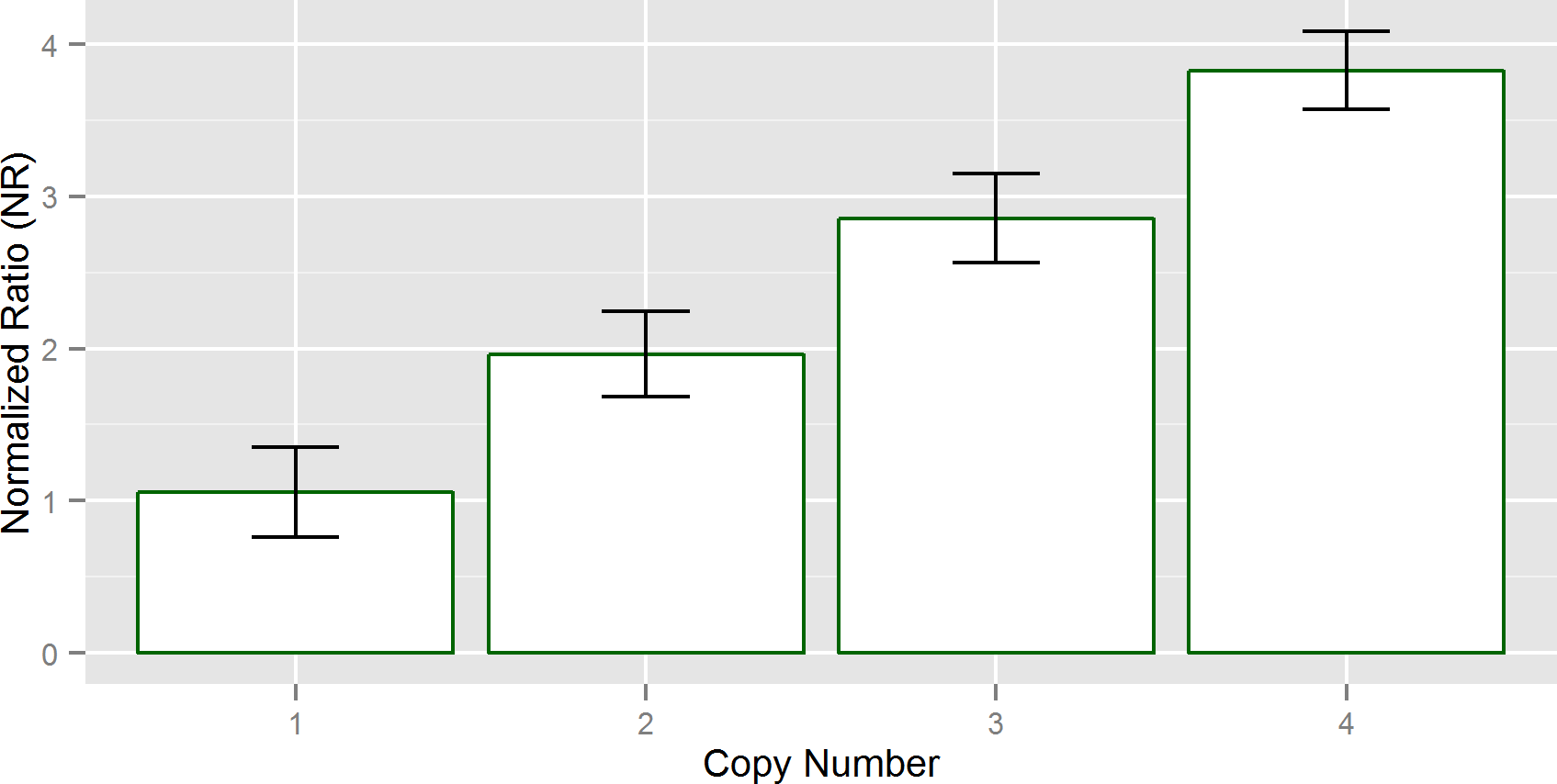

Supplement: S2 Fig — Shown is the average for each copy number (CN) state for 16 tested primers (Nr. 2–17 in S9 Table). NR mean and standard deviation (SD) are 1.057 ± 0.295, 1.964 ± 0.281, 2.855 ± 0.291 and 3.827 ± 0.255 for 1n, 2n, 3n, and 4n, respectively. See Materials and Methods for details. (TIFF) [file pone.0157711.s002.tiff]

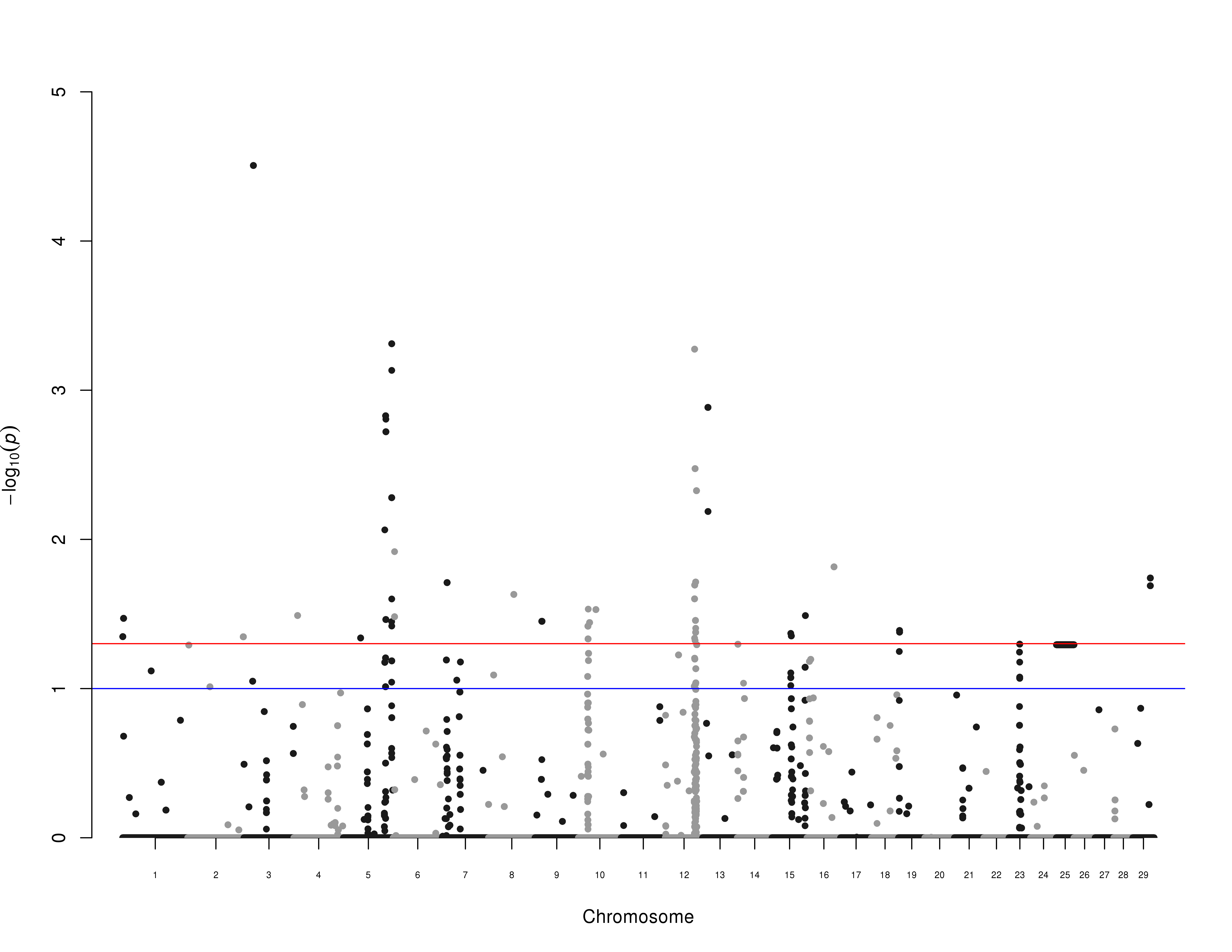

Supplement: S3 Fig — (TIFF) [file pone.0157711.s003.tiff]

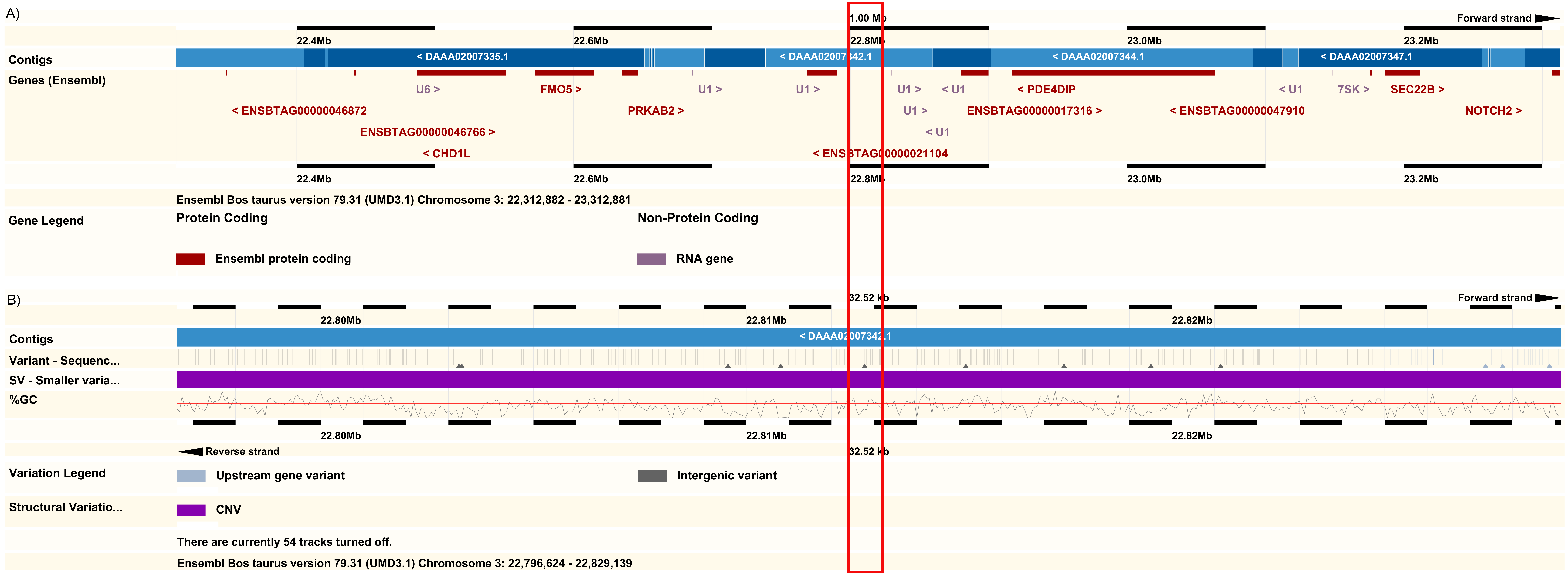

Supplement: S4 Fig — (A) Genomic positions. (B) Previously described CNVR in the Ensembl genome browser (http://www.ensembl.org/Bos_taurus/Location/View?r=3%3A22796624-22815905). (TIFF) [file pone.0157711.s004.tiff]

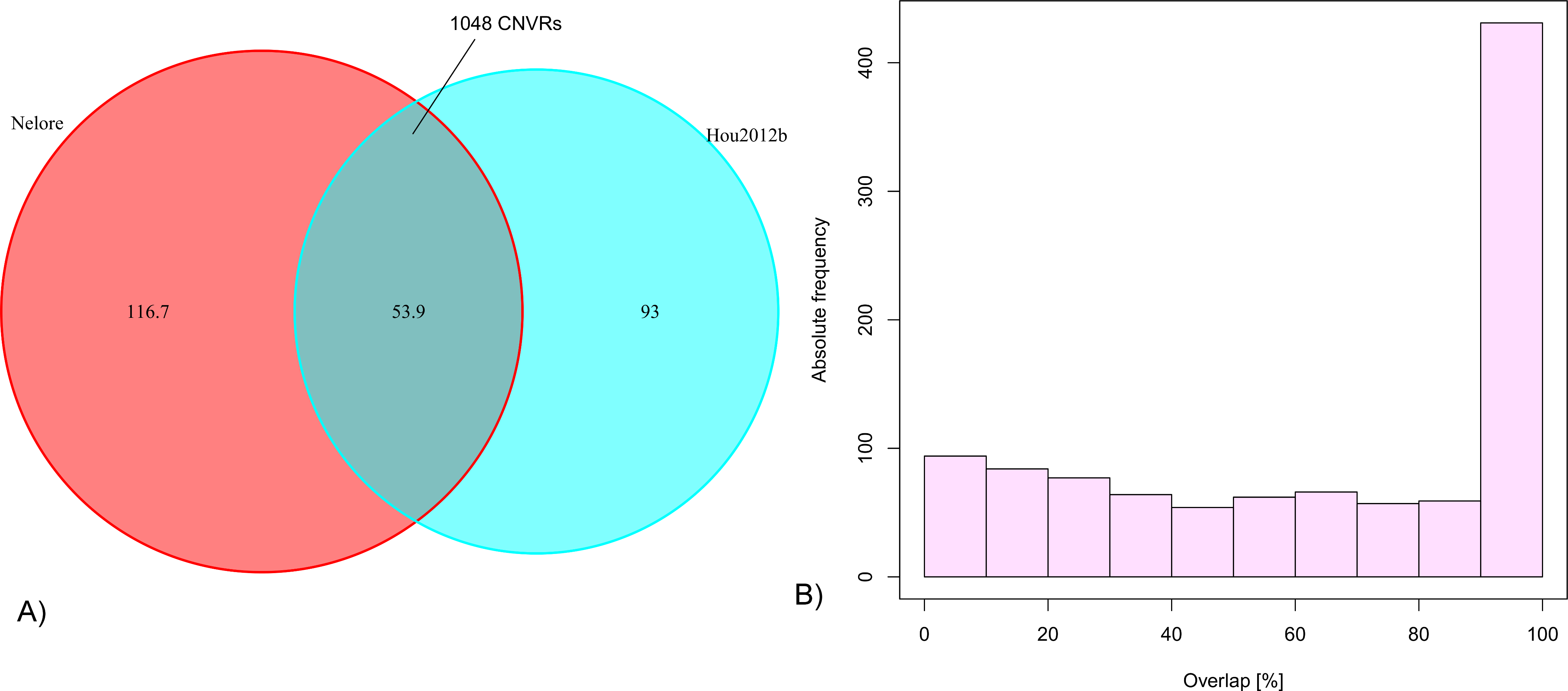

Supplement: S5 Fig — (A) The Venn diagram shows the genomic size in Mb that is overlapped. The overlapped fraction of 53.9 Mb corresponds to 1048 Nelore CNVRs. (B) For these 1048 CNVRs, the histogram shows the number of CNVRs (y-axis) overlapping with CNVRs from Hou et al. by the percentage shown on the x-axis. For example, genomic locations of >400 Nelore CNVRs overlap individually >90% with genomic locations of CNVRs from Hou et al. (TIFF) [file pone.0157711.s005.tiff]

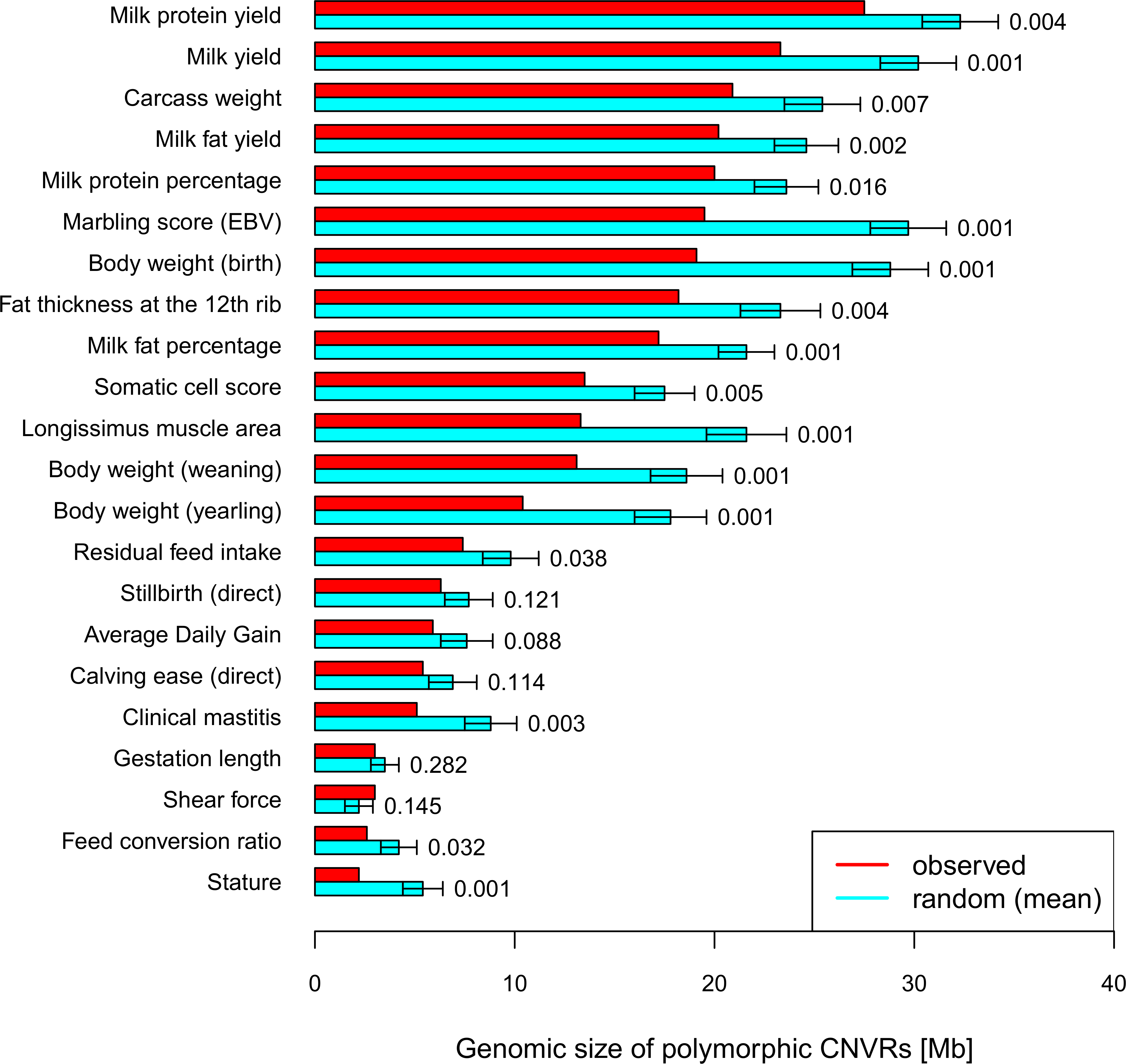

Supplement: S6 Fig — Depicted are the most frequently overlapped traits and the respective overlapped genomic size in Mb. (TIFF) [file pone.0157711.s006.tiff]
