# Supplementary material for: Genome-Wide Detection of CNVs and Their Association with Meat Tenderness in Nelore Cattle
Source: PLoS One. 2016 Jun 27;11(6):e0157711. doi: 10.1371/journal.pone.0157711 (PMC4922624; doi:10.1371/journal.pone.0157711)
Supplement: S2 Table — (PDF) [file pone.0157711.s008.pdf]

S2 Table. CNV studies in cattle.

References	Number of samples	Genome coverage (%)	% CNVRs in genes	Methods
Matukumalli et al. (2009) [1]	576	-	-	SNP-chip
Liu et al. (2010) [2]	90	1.07	67	CGH
Bae et al. (2010) [3]	265	-	-	SNP-chip
Fadista et al. (2010) [4]	20	0.68	30	CGH
Seroussi et al. (2010) [5]	912	-	15	SNP-chip
Hou et al. (2011) [6]	539	4.60	56	SNP-chip
Kijas et al. (2011) [7]	10	0.5	82	CGH
Zhan et al. (2011) [8]	1	0.16[a]	59	CNV-seq, SNP-chip, CGH
Stothard et al. (2011) [9]	2	0.13	33.3	CNV-seq
Bickhart et al. (2012) [10]	5	2.1	16.7	CNV-seq, CGH
Hou et al. (2012a) [11]	147	0.6	41	SNP-chip
Hou et al. (2012b) [12]	674	4.7	24	SNP-chip
Hou et al. (2012c) [13]	472	4.7	58	SNP-chip
Jiang et al. (2012) [14]	2047	0.91	9.65	SNP-chip
Cicconardi et al. (2013) [15]	2654	20	-	SNP-chip
Jiang et al. (2013) [16]	96	1.61	60	SNP-chip
Choi et al. (2013) [17]	3	0.29[b] - 0.63[c]	77.57[b] – 45,27[c]	CNV-seq

CGH: *comparative genomic hybridization* and CNV-seq: CNV regions obtained by DNA sequencing.
[a]Average of all methods.
[b]Hanwoo-Angus.
[c]Hanwoo-Holland.
[-] Not reported.

References

1. Matukumalli LK, Lawley CT, Schnabel RD, Taylor JF, Allan MF, et al. (2009) Development and Characterization of a High Density SNP Genotyping Assay for Cattle. PLoS One 4: 13.

2. Liu GE, Hou Y, Zhu B, Cardone MF, Jiang L, et al. (2010) Analysis of copy number variations among diverse cattle breeds. Genome Res 20: 693–703.

3. Bae JS, Cheong HS, Kim LH, NamGung S, Park TJ, et al. (2010) Identification of copy number variations and common deletion polymorphisms in cattle. BMC Genomics 11: 232.

4. Fadista J, Thomsen B, Holm L-E, Bendixen C (2010) Copy number variation in the bovine genome. BMC Genomics 11: 284.

5. Seroussi E, Glick G, Shirak A, Yakobson E, Weller JI, et al. (2010) Analysis of copy loss and gain variations in Holstein cattle autosomes using BeadChip SNPs. BMC Genomics 11: 673.

6. Hou Y, Liu GE, Bickhart DM, Cardone MF, Wang K, et al. (2011) Genomic characteristics of cattle copy number variations. BMC Genomics 12: 127.

7. Kijas JW, Barendse W, Barris W, Harrison B, McCulloch R, et al. (2011) Analysis of copy number variants in the cattle genome. Gene 482: 73–77.

8. Zhan B, Fadista J, Thomsen B, Hedegaard J, Panitz F, et al. (2011) Global assessment of genomic variation in cattle by genome resequencing and high-throughput genotyping. BMC Genomics 12: 557.

9. Stothard P, Choi J-W, Basu U, Sumner-Thomson JM, Meng Y, et al. (2011) Whole genome resequencing of black Angus and Holstein cattle for SNP and CNV discovery. BMC Genomics 12: 559.

10. Bickhart DM, Hou Y, Schroeder SG, Alkan C, Cardone MF, et al. (2012) Copy number variation of individual cattle genomes using next-generation sequencing. Genome Res 22: 778–790.

11. Hou Y, Bickhart DM, Chung H, Hutchison JL, Norman HD, et al. (2012) Analysis of copy number variations in Holstein cows identify potential mechanisms contributing to differences in residual feed intake. Funct Integr Genomics 12: 717–723.

12. Hou Y, Bickhart DM, Hvinden ML, Li C, Song J, et al. (2012) Fine mapping of copy number variations on two cattle genome assemblies using high density SNP array. BMC Genomics 13: 376.

13. Hou Y, Liu GE, Bickhart DM, Matukumalli LK, Li C, et al. (2012) Genomic regions showing copy number variations associate with resistance or susceptibility to gastrointestinal nematodes in Angus cattle. Funct Integr Genomics 12: 81–92.

14. Jiang L, Jiang J, Wang J, Ding X, Liu J, et al. (2012) Genome-wide identification of copy number variations in Chinese Holstein. PLoS One 7: e48732.

15. Cicconardi F, Chillemi G, Tramontano A, Marchitelli C, Valentini A, et al. (2013) Massive screening of copy number population-scale variation in Bos taurus genome. BMC Genomics 14: 124.

16. Jiang L, Jiang J, Yang J, Liu X, Wang J, et al. (2013) Genome-wide detection of copy number variations using high-density SNP genotyping platforms in Holsteins. BMC Genomics 14: 131.

17. Choi J-W, Lee K-T, Liao X, Stothard P, An H-S, et al. (2013) Genome-wide copy number variation in Hanwoo, Black Angus, and Holstein cattle. Mamm Genome 24: 151–163.
