# Supplementary material for: Genome-Wide Detection of CNVs and Their Association with Meat Tenderness in Nelore Cattle
Source: PLoS One. 2016 Jun 27;11(6):e0157711. doi: 10.1371/journal.pone.0157711 (PMC4922624; doi:10.1371/journal.pone.0157711)
Supplement: S5 Table — Shown is the number of CNVRs that overlap genes annotated in the bosTau6 assembly, QTLs from cattle QTLdb [45], Nelore MT-QTLs [63], and known CNVRs (S2, S3 and S10 Tables). Shown in brackets is the total genomic size in Mb that is overlapped. The observed overlap is shown in the 2nd column as compared to the mean overlap of random regions in the 3rd column (with standard deviation, SD). Random regions were sampled repeatedly (N = 1000) matching the CNVRs in size and chromosomal location. See S1 Fig for observed overlap types and Material and Methods for random sampling of genomic regions. The alternative tested, i.e. whether the observed overlap is less or greater than expected by chance, is shown in 4th column. The corresponding p-value (rounded to 3 decimal places) is shown in the 5th column. A p-value < 0.001 denotes that none of the 1000 permutations yielded an overlap as extreme as it has been observed. (PDF) [file pone.0157711.s011.pdf]

S5 Table. Overlap statistics. Shown is the number of CNVRs that overlap genes annotated in the bosTau6 assembly, QTLs from cattle QTLdb [1], Nelore MT-QTLs [2], and known CNVRs (S2, S3, and S10 Tables). Shown in brackets is the total genomic size in Mb that is overlapped. The observed overlap is shown in the 2nd column as compared to the mean overlap of random regions in the 3rd column (with standard deviation *sd*). Random regions were sampled repeatedly (*N*=1000) matching the CNVRs in size and chromosomal location. See *S1 Fig* for observed overlap types and *Material and Methods* for random sampling of genomic regions. The alternative tested, i.e. whether the observed overlap is less or greater than expected by chance, is shown in 4th column. The corresponding *p*-value (rounded to 3 decimal places) is shown in the 5th column. A *p*-value < 0.001 denotes that none of the 1000 permutations yielded an overlap as extreme as it has been observed.

**Overlap Category**	**Observed**	**Random Mean ± Sd**	**Alternative**	***P*-value**
All CNVRs				
Genes<br>(Mb)	1145<br>(38.2)	1265 ± 24<br>(47.1 ± 1.8)	less	< 0.001
Cattle QTLs<br>(Mb)	2520<br>(131.1)	2613 ± 13<br>(152.1 ± 3.3)	less	< 0.001
Nelore QTLs<br>(Mb)	109<br>(4)	124 ± 10<br>(6.2 ± 0.9)	less	0.057<br>(0.005)
Known CNVRs<br>(Mb)	1374<br>(64.7)	643 ± 21<br>(22.6 ± 1.4)	greater	< 0.001
Polymorphic CNVRs				
Genes<br>(Mb)	279<br>(16.1)	290 ± 10.5<br>(18.8 ± 1.2)	less	0.141<br>(0.012)
Cattle QTLs<br>(Mb)	471<br>(51.6)	499 ± 4<br>(60.3 ± 1.1)	less	< 0.001
Nelore QTLs<br>(Mb)	23<br>(1.1)	25 ± 5<br>(2.4 ± 0.76)	less	0.32<br>(0.024)
Known CNVRs<br>(Mb)	446<br>(39.5)	165 ± 10<br>(9.1 ± 1)	greater	< 0.001

References

1. Hu Z-L, Park CA, Wu X-L, Reecy JM (2013) Animal QTLdb: an improved database tool for livestock animal QTL/association data dissemination in the post-genome era. Nucleic Acids Res 41: D871–D879.

2. Tizioto PC, Decker JE, Taylor JF, Schnabel RD, Mudadu M, et al. (2013) Genome scan for meat quality traits in Nelore beef cattle. Physiol Genomics 45: 1012–1020.
