# Supplementary material for: Genome-Wide Detection of CNVs and Their Association with Meat Tenderness in Nelore Cattle
Source: PLoS One. 2016 Jun 27;11(6):e0157711. doi: 10.1371/journal.pone.0157711 (PMC4922624; doi:10.1371/journal.pone.0157711)
Supplement: S9 Table — (PDF) [file pone.0157711.s015.pdf]

**Primer number and name**	**Primer forward**	**Primer reverse**	**Amplicon (bp)**
**1** – BTF3 (control)	CACCCTGAGCAAAACCACTA	CAAACACTTTCACCATTACGG	165
**2 - Col IV – 1**	ACAGAGTCTTGACACTGAACCAC	TTCTTGGAGTAGGGGGTGAC	205
**3 - Col IV – 2**	TGCGGTTGGTAAAAAGTTCA	ATCAAGCAGGGATGTGGAGT	183
**4 - Ser/Thr**	TGACGAGAGGCTCACAGAAA	TCTTCAAAACGATGCCAAAT	224
**5 - Chr5_SNP1**	AACAACTGAAGTGACCAAGCA	CACACCCTCTCCAGCATTTA	125
**6 - Chr5_SNP2**	AGAGCCCCAGAGAACCACT	AGACGGGACCAGCAAGAA	106
**7 - Chr7_SNP1**	CCACTGGGAGACTGTTACGA	ATTGTGGCATTAGGGGAAAA	98
**8 -Chr7_SNP2**	CCCAGAACAAACAAAACAGC	CCAGCGACTCTGCTCATTTA	82
**9 - Chr9_SNP1**	CCACCCTACCTTTGCTGAGT	ATAAAAGGCTGGCAGAGGAA	145
**10 - Chr9_SNP2**	GCCCTCTCTGACCTCATCTC	CCTGACAGAGAAGCCAACAA	87
**11 - Chr10_SNP1**	GCAGGGAATAAGAACTGAGAGG	CTTAGCGGGCTGTAATCCAT	119
**12 - Chr10_SNP2**	AGGTCCTCAGGGGAGAAGAT	GCTGAAGCCACAAGACTATGA	105
**13 - Chr13_SNP1**	GTTTCCACCAACATTTCCAA	TAGACGCACTCCAGTCCAAC	98
**14 - Chr13_SNP2**	CCAGTTACAGGCATACAGTTCTTT	GGTGAAATGGAAAAGGCAGT	108
**15 - Chr15_SNP1**	AGGTTTATTGGAATGGCTGAC	CAACTGAGCGACTGAACTGAA	147
**16 - Chr15_SNP2**	CTTATGAGGCTGAAGTCAATGC	GGAACATTAGAGTTTGTCTCAGGAT	123
**17 - Chr25_SNP1**	TCCAAGTCTAAGGCTGCTGA	GATTTACCCCGCTATATTTGATGT	116
**18 - CNVR323**	CTGACTTGCTTTCACCTGGA	CGTCTTCGGGGAAACATTAT	122
