# Supplementary material for: Genome-Wide Detection of CNVs and Their Association with Meat Tenderness in Nelore Cattle
Source: PLoS One. 2016 Jun 27;11(6):e0157711. doi: 10.1371/journal.pone.0157711 (PMC4922624; doi:10.1371/journal.pone.0157711)
Supplement: S1 Text — (PDF) [file pone.0157711.s019.pdf]

Concerning to genes previously associated with cattle meat tenderness, the µ-calpain (*CAPN1* - ENSBTAG00000010230) and calpastatin (*CAST* - ENSBTAG00000000874) are key ones for cattle in general [1,2] and even particularly for the Nelore breed [3,4]. The remarkable influence in postmortem protein degradation, from *CAPN1* and *CAST*, can partially explain the association with meat tenderness [5]. However, none of our Nelore CNVRs have overlap with *CAPN1*/*CAST* genes. Likewise none of miRNAs genes, predicted to bind on *CAPN1*/*CAST* product transcripts, were overlapped by our Nelore CNVRs or even known CNVRs. Conversely, 3 known CNVRs were overlapped with *CAPN1* or *CAST* genes. One known CNVR overlapped with *CAPN1* gene is described for *Bos taurus coreanae* (Hanwoo) [6]. Two previously described CNVRs encompassing *CAST* gene, one for Holstein-Friesian dairy cattle (*Bos taurus taurus*) [7] and another in a study with 27 different cattle breeds including Holstein [8].

Heat shock proteins (HSPs) are involved with key processes in meat tenderness [9]. HSPs and other proteins are associated with Nelore meat tenderness [10]. However, none of our Nelore CNVRs, or known CNVRs coordinates, have overlap with source genes of these associated proteins (HSP27, HSP70, MLC2, alpha tropomyosin, beta-LG and cytochrome b-c1 subunit 1) [10]. It can indicate a lack of copy number events in those genomic regions. Similarly, Ensembl *Bos Taurus* annotated miRNAs with predicted binding sites inside to these 6 protein genes do not overlap our Nelore CNVRs. The exception was bta-miR-499 (ENSBTAG00000029949) miRNA gene, which is predicted to bind at alpha tropomyosin TPM1-201 transcript (ENSBTAT00000044796) are inside a CNVR in a lost state in Hanwoo breed (*Bos taurus coreanae*) and gain state in Angus breed (*Bos taurus taurus*) [11]. The tenderness score is higher for Hanwoo when compared with Angus [12].

References

1. Casas E, White SN, Riley DG, Smith TPL, Brenneman RA, Olson TA, et al. Assessment of single nucleotide polymorphisms in genes residing on chromosomes 14 and 29 for association with carcass composition traits in Bos indicus cattle. J Anim Sci. 2005;83: 13–19.

2. Tait RG, Shackelford SD, Wheeler TL, King DA, Casas E, Thallman RM, et al. μ-Calpain, calpastatin, and growth hormone receptor genetic effects on pre-weaning performance, carcass quality traits, and residual variance of tenderness in Angus cattle selected to increase minor haplotype and allele frequencies. J Anim Sci. 2014 Jan 7;92: 456–466.

3. Pinto LFB, Ferraz JBS, Meirelles FV, Eler JP, Rezende FM, Carvalho ME, et al. Association of SNPs on CAPN1 and CAST genes with tenderness in Nellore cattle. Genet Mol Res. 2010;9(3): 1431–1442.

4. Curi RA, Chardulo LAL, Mason MC, Arrigoni MDB, Silveira AC, de Oliveira HN. Effect of single nucleotide polymorphisms of CAPN1 and CAST genes on meat traits in Nellore beef cattle (Bos indicus) and in their crosses with Bos taurus. Anim Genet. 2009 Aug;40(4): 456–462.

5. Tizioto PC, Gromboni CF, Nogueira ARDA, de Souza MM, Mudadu MDA, Tholon P, et al. Calcium and potassium content in beef: influences on tenderness and associations with molecular markers in Nellore cattle. Meat Sci. 2014 Jan;96(1): 436–440.

6. Bae JS, Cheong HS, Kim LH, NamGung S, Park TJ, Chun J-Y, et al. Identification of copy number variations and common deletion polymorphisms in cattle. BMC Genomics. 2010 Jan;11: 232.

7. Zhan B, Fadista J, Thomsen B, Hedegaard J, Panitz F, Bendixen C. Global assessment of genomic variation in cattle by genome resequencing and high-throughput genotyping. BMC Genomics. 2011 Jan;12(1): 557.

8. Hou Y, Bickhart DM, Hvinden ML, Li C, Song J, Boichard D a, et al. Fine mapping of copy number variations on two cattle genome assemblies using high density SNP array. BMC Genomics. 2012 Jan;13: 376.

9. D'Alessandro A, Marrocco C, Rinalducci S, Mirasole C, Failla S, Zolla L. Chianina beef tenderness investigated through integrated Omics. J Proteomics. 2012 Jul 19;75(14): 4381–4398.

10. Carvalho ME, Gasparin G, Poleti MD, Rosa AF, Balieiro JCC, Labate CA, et al. Heat shock and structural proteins associated with meat tenderness in Nellore beef cattle, a Bos indicus breed. Meat Sci. 2014 Mar;96(3): 1318–1324.

11. Choi J-W, Lee K-T, Liao X, Stothard P, An H-S, Ahn S, et al. Genome-wide copy number variation in Hanwoo, Black Angus, and Holstein cattle. Mamm Genome. 2013 Apr;24(3-4): 151–163.

12. Van Ba H, Ryu KS, Lan NTK, Hwang I. Influence of particular breed on meat quality parameters, sensory characteristics, and volatile components. Food Sci Biotechnol. 2013 Jun 30;22(3): 651–658.
